# Three-Month Incidence of Venous Thromboembolism in Patients Who Underwent Neurological Surgeries

**DOI:** 10.3390/jcm14020552

**Published:** 2025-01-16

**Authors:** Petnumnueng Ponsumritchok, Praepattra Chaijaroen, Tin Ayurag, Nattaphan Siritikul, Piangrawee Niprapan, Nonthakorn Hantrakun, Jirapong Vongsfak, Chatree Chai-Adisaksopha

**Affiliations:** 1 Faculty of Medicine, Chiang Mai University, Chiang Mai 50200, Thailand; 2 Division of Hematology, Department of Internal Medicine, Faculty of Medicine, Chiang Mai University, Chiang Mai 50200, Thailand; 3 Division of Neurosurgery, Clinical Surgical Research Center, Department of Surgery, Faculty of Medicine, Chiang Mai University, Chiang Mai 50200, Thailand

**Keywords:** incidence, VTE, neurological surgery patients

## Abstract

**Background/Objectives**: The incidences of venous thromboembolism (VTE) in patients undergoing neurological surgeries vary. The objectives were to assess the incidence and risk factors of VTE, bleeding and all-cause mortality in patients undergoing neurological surgery. **Methods**: This retrospective cohort study was conducted at a single center, a university-based hospital in Thailand. Inclusion criteria comprised patients aged 15 years or older who were admitted for elective or emergency neurosurgery. Patients with preoperative VTE diagnosed within three months or a history of anticoagulant use were excluded. Outcomes measured included the 90-day incidences of VTE, any bleeding, major bleeding, and mortality. **Results**: Between January 2021 and December 2022, a total of 626 patients were included. The mean age was 50.21 ± 17.37 years, and 55.27% were males. Thromboprophylaxis was administered to 86 patients (13.74%, 95% CI 11.14–16.69). Fourteen patients were confirmed to have symptomatic VTE, resulting in an incidence of 2.24%, with a 95% confidence interval (CI) of 1.23–3.72. Patients aged ≥75 years (HR 4.53; 95% CI 1.25–16.38; *p* = 0.021), those with cancer (HR 8.51; 95% CI 2.95–24.60, *p* <0.001), and those experiencing postoperative paraparesis/paralysis (HR 3.26; 95% CI 1.12–9.45; *p* = 0.030) were associated with an increased risk of postoperative VTE. Fifty-three patients (8.47%, 95% CI 6.41–10.93) experienced any bleeding, with 23 patients (3.67%, 95% CI 2.34–5.46) having major bleeding. The incidence of postoperative mortality was 6.55%, with a 95% CI of 4.74–8.78. **Conclusions**: This study revealed that elderly patients, those with cancer or those experiencing postoperative paraparesis/paralysis were at higher risk of VTE. These patients were likely to benefit from VTE prophylaxis.

## 1. Introduction

Venous thromboembolism (VTE) is composed of deep vein thrombosis (DVT) and pulmonary embolism (PE). These complications are found to have increased prevalence in neurosurgical patients and would lead to increased mortality rates [1].

In the general population, the prevalence of DVT ranges from 45 to 117 in 100,000 individuals and the prevalence in the male population (130 in 100,000) is higher than that in the female population (110 in 100,000) across all age groups [2]. Studies of DVT prevalence in different ethnic groups show that African-Americans have the highest prevalence compared to Caucasians and Latin-Americans, with Asians and Pacific Islanders having the lowest prevalence among all ethnic groups [3].

The pathophysiology of VTE can be explained by Virchow’s triad including venous stasis, vascular injury, and hypercoagulability [4]. Consequently, surgical patients are at risk of developing VTE. The risk of VTE in surgical patients depends on age, sex, patients’ presenting condition, co-morbid diseases and type of surgery [5]. Neurosurgery is one of the surgical procedures carrying the highest risk of postoperative VTE [6].

On the one hand, neurosurgical patients who have not been given any thromboprophylaxis have a prevalence of DVT ranging from 0 to 34% [2]. On the other hand, patients who have been given thromboprophylaxis have a prevalence of 3 to 16% [7,8] as diagnosed with doppler ultrasound procedures, and 1 to 4% that are shown to have symptoms of DVT [2]. PE is a severe complication that could be a sequel to DVT and has high disability and mortality rates. Neurosurgical patients have an approximate risk of PE at 0 to 5%, associated with a mortality rate of 9 to 50% [1].

Variations in the prevalence of venous thromboembolism (VTE) exist among neurosurgical patients, influenced by race and the specific type of neurosurgical procedure. The risk of VTE in neurosurgical patients is related to the type and location of the surgery. Data from the Analysis of the National Surgical Quality Improvement Program database reported that the risk of DVT was 3.4% following cranial surgeries and 1.1% following spinal surgeries [9,10]. In addition, patients who underwent neurosurgery for tumors had a 2–10% risk of VTE, whereas those who underwent surgery for subarachnoid hemorrhage had a risk of VTE ranging from 3.5–18% [11,12,13,14,15]. Among patients with traumatic brain injury, the risk of VTE increased threefold to fourfold compared with patients without traumatic brain injury, regardless of the timing of thromboprophylaxis initiation [16].

Consequently, the role of thromboprophylaxis in this patient population remains a subject of controversy. This study aimed to examine the incidence of postoperative VTE in Thai patients undergoing neurological surgeries. 

## 2. Materials and Methods

### 2.1. Patients

This study was a retrospective review of the cohort database of patients who underwent elective or emergency neurosurgery at Maharaj Nakorn Chiang Mai Hospital—a university hospital in Northern Thailand—between January 2021 and December 2022. We included all patients who were 15 years of age or older who were admitted to the hospital for elective or emergency neurosurgery. Patients with preoperative VTE diagnosed within 3 months or history of anticoagulant use were excluded from the study. Patients for whom significant data were missing, including sex, age, duration of admission, duration of surgery date to outcome date and survival, were also excluded. Patients who were admitted outside the neurosurgery ward for any reason were also excluded.

### 2.2. Variables and Outcomes of Interest

The parameters which were recorded in this study included sex, age, duration of admission, underlying diseases, smoking status, concomitant medication use including aspirin and anticoagulants, previous venous or arterial thromboembolism, previous chemotherapy or radiotherapy within 3 months, weight, height, body mass index (BMI), diagnosis, type of neurosurgery, duration of surgery, American Society of Anesthesiologist (ASA) classification, transfusion during surgery, postoperative immobilization, pre-operative laboratory investigations and thromboprophylaxis. Immobilization was classified as patients who cannot walk independently after surgery. Mechanical thromboprophylaxis was defined as the use of intermittent pneumatic compression devices. Pharmacologic thromboprophylaxis included the use of prophylactic doses of low-molecular-weight heparin, unfractionated heparin, direct oral anticoagulants, or aspirin. Both mechanical and pharmacological thromboprophylaxis were initiated within 24 h after surgery. Thromboprophylaxis was continued until patients were discharged from the hospital.

The primary outcome of this study was the occurrence of symptomatic VTE within 90 days after surgery. Diagnosis of VTE was based on the objective evidence of thrombosis by imaging, which was either doppler ultrasonography or computed tomography (CT) scan angiography. The secondary outcomes were any bleeding, major bleeding and death within 90 days after surgery. Major bleeding was defined according to the International Society on Thrombosis and Haemostasis (ISTH) criteria [17]. The definition encompasses fatal bleeding, bleeding in a critical organ (such as intracranial or intraspinal) or bleeding leading to a decrease in hemoglobin level of 2.0 g/dL [17]. For the 90-day follow-up protocol, after patients were included in the study based on predefined inclusion and exclusion criteria, we conducted a comprehensive review of their medical records. This included outpatient and emergency visit records, radiological reports, and physician notes to identify clinical outcomes, specifically symptomatic VTE, bleeding and all-cause mortality, up to 90 days post-surgery.

### 2.3. Sample Size

The estimated sample size was calculated using a one-sample proportion test in Stata version 16 software with the desired power of 80% and the alpha level of 0.05. Considering an assumed incidence of VTE in neurosurgery patients at 7%, we determined that a sample size of 626 would be required to achieve a 2% absolute precision with 95% confidence.

### 2.4. Statistical Analysis

Patient characteristics were presented as descriptive statistics including frequencies with percentages for categorical data, mean with standard deviation (SD) for parametric data, and median with interquartile range (IQR) for continuous nonparametric data. Incidences of VTE, bleeding and mortality possibilities were analyzed through a survival curve analysis using the Kaplan–Meier (KM) method. In addition, Cox proportional hazards regression analysis was used to identify univariable and multivariable factors associated with outcomes. Variables demonstrating an association with VTE, bleeding, and mortality at a significance level of *p* ≤ 0.05 in univariable analysis were incorporated into the multivariable analysis using the backward stepwise method. Statistical analyses were conducted using Stata Statistical Software: Release 17. (College Station, TX: StataCorp LLC). Approval for this research was granted by the ethical committee of Chiang Mai University, Faculty of Medicine (No.128/2023).

## 3. Results

**Figure 1:**
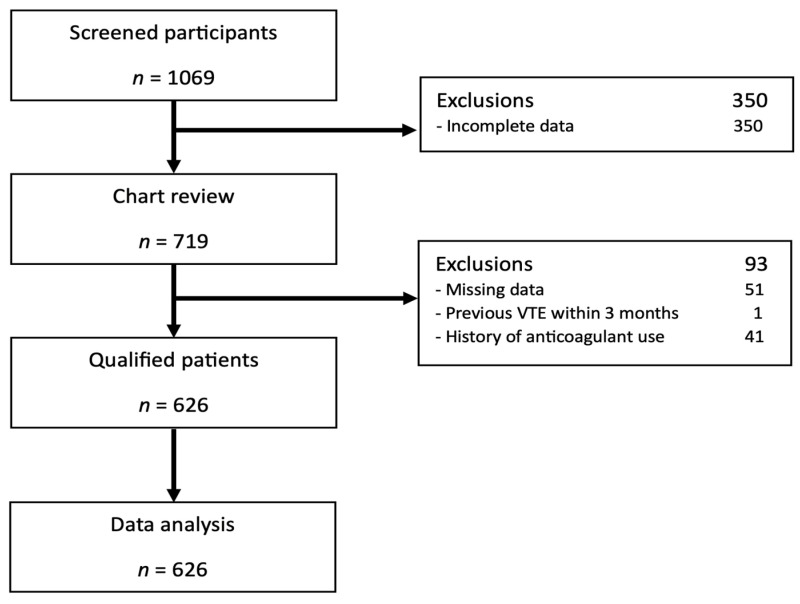
Flow diagram.

Between January 2021 and December 2022, a total of 1096 patients who underwent neurosurgeries were screened for eligibility. Four hundred and forty-three patients were subsequently excluded (Figure 1). The reasons for exclusion are shown in Figure 1. Consequently, the analysis included a total of 626 patients.

Table 1 demonstrates the baseline and demographic data of the study participants. The patients were categorized into two groups: VTE group (*n* = 14) and non-VTE group (*n* = 612). The mean age of the VTE group was significantly higher than that of the non-VTE group, with a mean of 59.50 (SD = 16.39) years versus 50.00 (SD = 17.34) years, respectively. A higher proportion of patients in the VTE group had cancer at the time of neurosurgeries compared to those in the non-VTE group (57.14% versus 12.91%). No statistically significant differences were observed in terms of co-morbidities, concurrent medications and types of neurological surgeries between the two groups.

The median duration of surgery was significantly higher in the VTE group as compared to the non-VTE group (3.00 h [IQR: 2, 4.34] in the VTE group and 1.95 h [IQR: 1.25, 3] in the non-VTE group). Post-operative paraparesis/paralysis were more prevalent among patients in the VTE group (57.14%) than in the non-VTE group (28.76%).

### 3.1. Thromboprophylaxis

Table 2 shows thromboprophylaxis in patients who underwent neurological surgeries. Thromboprophylaxis was administered to 86 patients (13.74%, 95% CI 11.14–16.69). Among these, 58 patients (9.26%) received mechanical prophylaxis, while 44 patients (7.01%) received pharmacoprophylaxis and 16 patients (2.56%) received both mechanical prophylaxis and pharmacoprophylaxis. Aspirin was utilized in 65.91% of patients who received pharmacoprophylaxis, followed by low-molecular-weight-heparin at 40.91%. As shown in Table 2, 29 patients received aspirin, of whom three continued aspirin use prior to surgery. Among these, only one patient discontinued aspirin 7 days before surgery. To clarify the details regarding the use of aspirin during surgery, we have added a paragraph to the Results section.

### 3.2. Post-Operative Venous Thromboembolism

Fourteen patients were confirmed to have symptomatic VTE within 90 days after neurosurgeries, giving an incidence of 2.24%, 95% confidence interval (CI) 1.23–3.72. The median time of VTE occurrence was 12 days. There were four cases of DVT (0.64%), eight cases of PE (1.28%) and two cases of both DVT and PE (0.32%).

There were 13 out of 540 patients (2.40%) who did not receive VTE prophylaxis and had VTE. On the other hand, 1 out of 102 patients (0.98%) received mechanical prophylaxis and had VTE. None of the patients who received pharmacological prophylaxis had VTE.

**Figure 2:**
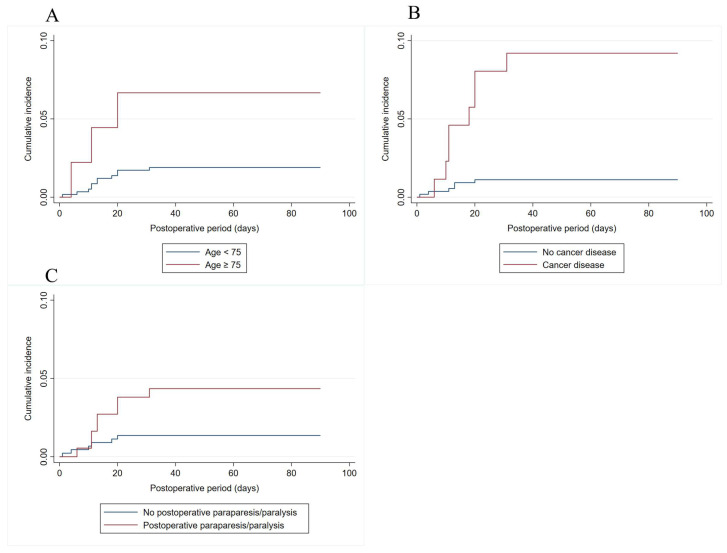
Risk of developing ninety-day post-operative VTE when patients were classified according to age (**A**), cancer (**B**) and postoperative paraparesis/paralysis status (**C**).

Table 3 demonstrates the results of multivariable Cox regression analysis for VTE. Patients with age ≥ 75 years (HR 4.53; 95% CI 1.25–16.38; *p* = 0.021), those with cancer (HR 8.51; 95% CI 2.95–24.60, *p* < 0.001), and those experiencing postoperative paraparesis/paralysis (HR 3.26; 95% CI 1.12–9.45; *p* = 0.030) were associated with an increased risk of post-operative VTE. However, multivariable Cox regression analysis did not show a significant association between pharmacological prophylaxis and the decreased risk of VTE. Figure 2 shows the risk of developing post-operative VTE when patients were classified according to age, cancer and postoperative paraparesis/paralysis status.

### 3.3. Post-Operative Bleeding

Fifty-three patients (8.47%, 95% CI 6.41–10.93) experienced any bleeding within 90 days after neurosurgeries. Of these, 23 patients (3.67%, 95% CI 2.34–5.46) had major bleeding.

Table 3 demonstrates the results of multivariable Cox regression analysis for post-operative bleeding. Patients with age ≥ 60 years were associated with higher risk of bleeding (HR 2.08, 95% CI 1.19–3.65, *p* = 0.010), as well as the presence of diabetes mellitus (HR 1.95; 95% CI 1.00–3.79, *p* = 0.049), ASA classification ≥ 4 (HR 2.30, 95% CI 1.13–4.67, *p* = 0.021). Nevertheless, the receipt of thromboprophylaxis did not significantly increase risk of any bleeding with HR 2.11, 95% CI 0.95–4.71, *p* = 0.067. Similarly, increase in age was associated with increased risk of major bleeding with HR 1.02, 95% CI 1.00–1.05, *p* = 0.049, as well as ASA classification ≥ 4 (HR 4.23, 95% CI 1.65–10.90, *p* = 0.003).

### 3.4. Post-Operative All-Cause Mortality

Forty-one patients died within 90 days after the surgeries, giving an incidence of post-operative morality of 6.55%, 95% CI 4.74–8.78.

Table 3 demonstrates the results of multivariable Cox regression analysis for post-operative mortality. Presence of chronic kidney disease was associated with increased risk of post-operative mortality with HR of 8.89, 95% CI 2.89–27.38, *p* < 0.001, along with other factors including cancer (HR 6.11, 95% CI, 2.32–16.07, *p* < 0.001), head injury (HR 4.27, 95% CI 1.65–10.44, *p* < 0.001), transfusion during surgery (HR 2.69, 95% CI 1.26–5.74, *p* = 0.017), postoperative paraparesis/paralysis (HR 5.55, 95% CI 2.64–11.66, *p* < 0.001), and post-operative bleeding (HR 2.23; 95% CI 1.09–4.59, *p* = 0.029). Patients who had duration of surgery ≥ 45 min were associated with lower risk of mortality (HR 0.23, 95% CI 0.08–0.63, *p* = 0.004).

## 4. Discussion

In this study, we assessed the incidence and risk factors associated with patients who underwent neurological surgeries. The 90-day incidence of postoperative symptomatic VTE was observed at 2.24%. Additionally, incidences of any bleeding and death were observed at 8.31% and 6.55%, respectively.

The study was conducted in Malaysia, analyzed 320 patients and reported an overall incidence of DVT of 10.3% [18]. A more recent retrospective study involving 350 Thai patients who underwent neurological surgery indicated the incidence of VTE of 7.4% [19]. Liu et al. conducted a retrospective analysis of elective patients undergoing resection of malignant brain tumors in China [20], where out of 456 patients, 24.6% experienced postoperative thrombosis-related complications [20]. Among these, 18.4% had DVT, while the remaining patients suffered from cerebral ischemia [20].

The variation in the incidence of VTE across these studies may be attributed to differences in patient characteristics, the implementation of thromboprophylaxis, the type of neurosurgery performed, or the methods used to assess outcomes. A prospective study focusing specifically on the Asian population is urgently needed to provide precise insights into VTE complications following neurosurgery.

In this present study, age ≥ 75 years, diagnosis of cancer, and postoperative paraparesis/paralysis were independent risk factors for postoperative VTE. However, smoking, obesity, sex, diabetes, hypertension, dyslipidemia, and history of venous or arterial thrombosis were not associated with increased risk of VTE. Consistent with our findings, previous studies on neurosurgical patients undergoing craniotomy have indicated a significant association between older age and increased VTE risk [12,21]. Aishima et al. conducted a retrospective study in Japan involving 419 patients who underwent brain tumor surgery, revealing that malignant histology and preoperative paresis were independent risk factors for VTE [22]. Similarly, a retrospective study in the United States, focusing on patients undergoing elective brain tumor surgery, demonstrated that certain tumor types, including high-grade oligodendroglioma, high-grade glioma, lymphoma, and lesions of metastatic or mixed pathology, were associated with a higher risk of postoperative VTE [23]. Another study, which included Thai patients who underwent brain tumor surgery, indicated that new-onset postoperative motor deficit and diabetes mellitus were VTE-associated risk factors [24].

While the risk factors for VTE in patients undergoing neurosurgery share similarities with those in the general population, such as older age or immobilization, this specific patient population presents unique characteristics, specifically malignant brain tumors and postoperative paresis, which may not be present in other patients. Recognizing these distinctive factors is important, as individuals at risk may require tailored VTE prevention procedures to reduce the risk of thrombotic complications following surgery.

This study identified independent risk factors of overall and major bleeding, which included older age, diabetes mellitus and ASA classification of 4 or higher. These findings align with previous studies that reported higher risk of bleeding or blood transfusion in patients with higher ASA classification and those with diabetes [25,26]. Pharmacoprophylaxis with anticoagulants may increase the risk of bleeding [27]. However, in this present study, the risk of overall or major bleeding did not increase among patients who were given pharmacoprophylaxis.

This study demonstrated that chronic kidney disease, cancer, head injury, transfusion during surgery, postoperative paraparesis/paralysis, and bleeding increased risk of all-cause mortality after surgery. These findings are consistent with previous research which reported that higher mortality was observed among patients with chronic kidney disease, disseminated cancer, postoperative dependent status, and bleeding disorders [28].

The American Society of Hematology 2019 guidelines recommend providing VTE prophylaxis with mechanical methods in patients undergoing major neurosurgical procedures [6]. However, we observed that only 13.74% of patients in this cohort received VTE prophylaxis. The adherence to VTE prophylaxis in neurosurgery patients has been reported to vary from 30% to 52%. Apart from poor protocol adherence, a recent survey among neurological patients revealed a lack of awareness regarding VTE [29,30,31]. To enhance VTE prevention, it is crucial to improve adherence to VTE prophylaxis protocols in patients and provide training on VTE knowledge to raise awareness among them.

We observed that patients who received mechanical thromboprophylaxis had a numerically lower risk of VTE compared to those who did not receive thromboprophylaxis. More importantly, none of the patients who received pharmacoprophylaxis developed post-operative VTE. However, after adjusting for potential confounders using multivariable Cox regression analysis, no significant association was found between pharmacoprophylaxis and the risk of VTE. This finding aligns with previous evidence suggesting that pharmacoprophylaxis has a small, possibly negligible effect on symptomatic proximal DVT (relative risk 0.84, 95% CI 0.03–0.84) [6].

The use of aspirin for post-surgical prophylaxis has been extensively studied in orthopedic patients. The American Society of Hematology guidelines on the prevention of venous thromboembolism in surgical hospitalized patients recommends the use of aspirin or anticoagulants in patients undergoing total hip or total knee arthroplasty [6]. However, evidence supporting the use of aspirin for thromboprophylaxis in neurosurgical patients is extremely limited. We observed no incidents of VTE among patients who received aspirin perioperatively in this study. This finding highlights the potential role of aspirin as a thromboprophylactic agent, which warrants further investigation through dedicated clinical studies.

Based on the results of this study, patients at high risk of VTE, including those who were 75 years or older, had active cancer, or had paraplegia/paraparesis after neurosurgery, are the ones who could potentially benefit from VTE prophylaxis.

The strength of this study lies in the systematic inclusion of patients using stringent criteria, ensuring a comprehensive and accurate review of all charts rather than relying on administrative data or diagnostic coding. This approach guarantees the precision of clinical variables, laboratory data, and events.

However, certain limitations need to be acknowledged. Firstly, the collection of outcomes was limited to a 90-day period, and events occurring beyond this cut-off would not be captured. Consequently, comparisons with studies featuring longer follow-up periods are constrained. Secondly, all patients in this study were of Thai ethnicity. As VTE is less common in the Asian population, the generalizability of the findings to patients of other ethnicities may be limited. Thirdly, we did not include inherited thrombophilia as a potential risk factor of VTE in this study. Lastly, this study exclusively examines symptomatic VTE cases, which may not fully represent the entire spectrum of VTE occurrences. Future research incorporating surveillance Doppler ultrasound diagnostic methods may provide a more comprehensive understanding of VTE by identifying asymptomatic cases in neurological surgery patients.

## 5. Conclusions

This study revealed that, among patients who underwent neurological surgery, individuals in the elderly age group, those diagnosed with cancer, or those experiencing postoperative paraparesis/paralysis faced a higher risk of VTE. These patients were likely to benefit from VTE prophylaxis.

## Figures and Tables

**Table 1:** Demographic data and baseline characteristics of enrolled patients.

Characteristic	Total (*n* = 626)	Non-VTE (*n* = 612)	VTE (*n* = 14)	*p*-Value
**Age (year), mean ±SD**	50.21±17.37	50.00 ±17.34	59.50 ±16.39	**0.043**
**Sex, *n* (%)**				0.887
Male	346 (55.27)	338 (55.23)	8 (57.14)	-
BMI (kg/m^2^), mean ±SD	23.60 ±4.05	23.57 ±4.05	24.88 ±3.88	0.231
**Comorbidity, *n* (%)**				
Diabetes mellitus	76 (12.14)	74 (12.09)	2 (14.29)	0.683
Hypertension	216 (34.5)	210 (34.31)	6 (42.86)	0.506
Dyslipidemia	132 (21.09)	128 (20.92)	4 (28.57)	0.508
Smoking	147 (23.48)	144 (23.53)	3 (21.43)	0.999
History of venous thromboembolism	1 (0.16)	1 (0.16)	0 (0.00)	0.999
History of arterial thromboembolism	19 (3.04)	18 (2.94)	1 (7.14)	0.353
Chronic kidney disease	17 (2.72)	17 (2.78)	0 (0.00)	0.999
**Current medication, *n* (%)**				
Concomitant aspirin use	42 (6.71)	40 (6.54)	2 (14.29)	0.240
History of chemotherapy within 3 months	6 (0.96)	6 (0.98)	0 (0.00)	0.999
History of radiotherapy within 3 months	3 (0.48)	2 (0.33)	1 (7.14)	0.066
**Cancer, *n* (%)**	87 (13.90)	79 (12.91)	8 (57.14)	**<0.001**
**ASA classification, *n* (%)**				
ASA 1	25 (3.99)	25 (4.08)	0 (0.00)	0.999
ASA 2	216 (34.50)	210 (34.31)	6 (42.86)	0.506
ASA 3	327 (52.24)	322 (52.61)	5 (35.71)	0.281
ASA 4	52 (8.31)	49 (8.01)	3 (21.43)	0.102
ASA 5	6 (0.96)	6 (0.98)	0 (0.00)	0.999
ASA 6	0 (0.00)	0 (0.00)	0 (0.00)	-
**Duration of surgery, median [p25, p75]**	3 [2, 4.33]	3 [2, 4.34]	1.95 [1.25, 3]	**0.022**
**Type of neurosurgery, *n* (%)**				
Head injury	201 (32.11)	196 (32.03)	5 (35.71)	0.776
Tumor	207 (33.07)	201 (32.84)	6 (42.86)	0.431
Vascular	83 (13.26)	83 (13.56)	0 (0.00)	0.235
Hydrocephalus	22 (3.51)	21 (3.43)	1 (7.14)	0.397
Infection	10 (1.60)	9 (1.47)	1 (7.14)	0.204
Miscellaneous	16 (2.56)	16 (2.61)	0 (0.00)	0.999
Spine	41 (6.55)	41 (6.70)	0 (0.00)	0.615
Functional surgery	11 (1.76)	11 (1.80)	0 (0.00)	0.999
Peripheral nerves	1 (0.16)	1 (0.16)	0 (0.00)	0.999
Ventriculostomy, lumbar drain etc.	34 (5.43)	33 (5.39)	1 (7.14)	0.546
**Transfusion during surgery, *n* (%)**	202 (32.27)	198 (32.35)	4 (28.57)	0.999
Pack red cell, *n* (%)	192 (30.67)	189 (30.88)	3 (21.43)	0.567
Platelet, *n* (%)	53 (8.47)	50 (8.17)	3 (21.43)	0.107
Postoperative assisted ventilation, *n* (%)	340 (54.31)	331 (54.08)	9 (64.29)	0.590

Abbreviations: VTE; venous thromboembolism., Statistical significant *p*-values is in bold.

**Table 2:** Thromboprophylaxis in patients who underwent neurological surgery.

Thromboprophylaxis	Total (*n* = 626)	Non-VTE (*n* = 612)	VTE (*n* = 14)	*p*-Value
**None, *n* (%)**	540 (86.26)	527 (86.11)	13 (92.86)	0.525
**Mechanical, *n* (%)**	58 (9.26)	57 (9.31)	1 (7.14)	0.076
**Pharmacoprophylaxis, *n* (%)**	44 (7.01)	44 (7.19)	0	**<0.050**
Aspirin, *n* (%)	29 (65.91)	29 (65.91)	0	-
Low-molecular-weight heparin, *n* (%)	18 (40.91)	18 (40.91)	0	-

Abbreviations: VTE; venous thromboembolism, Statistical significant *p*-values is in bold.

**Table 3:** Multivariable Cox regression analysis of post-operative venous thromboembolism, bleeding and all-cause mortality.

Variables	Hazard Ratio	95% Confidence Interval	*p*-Value
**Venous thromboembolism**			
Age ≥ 75 years	4.53	1.25–16.38	**0.021**
Cancer	8.51	2.95–24.60	**<0.001**
Postoperative paraparesis/paralysis	3.26	1.12–9.45	**0.030**
**Overall bleeding**			
Age ≥ 60 years	2.08	1.19–3.65	**0.010**
Diabetes mellitus	1.95	1.00–3.79	**0.049**
ASA ≥ 4	2.30	1.13–4.67	**0.021**
Pharmacoprophylaxis	2.11	0.95–4.71	0.067
**Major bleeding**			
Age (each year increase)	1.02	1.00–1.05	**0.049**
ASA ≥ 4	4.23	1.65–10.90	**0.003**
**All-cause mortality**			
Chronic kidney disease	8.89	2.89–27.38	**<0.001**
Cancer disease	6.11	2.32–16.07	**<0.001**
Head injury	4.27	1.75–10.44	**<0.001**
Duration of surgery ≥ 45 min	0.23	0.08–0.63	**0.004**
Transfusion during surgery	2.69	1.26–5.74	**0.017**
Postoperative paraparesis/paralysis	5.55	2.64–11.66	**<0.001**
Any bleeding	2.23	1.09–4.59	**0.029**

Abbreviations: ASA; American Society of Anesthesiologists, Statistical significant *p*-values is in bold.

## Data Availability

No new data were created or analyzed in this study.

## References

[1] Hamilton M.G., Hull R.D., Pineo G.F. (1994). Venous thromboembolism in neurosurgery and neurology patients: A review. Neurosurgery.

[2] Shaikhouni A., Baum J., Lonser R.R. (2018). Deep Vein Thrombosis Prophylaxis in the Neurosurgical Patient. Neurosurg. Clin. N. Am..

[3] Keenan C.R., White R.H. (2007). The effects of race/ethnicity and sex on the risk of venous thromboembolism. Curr. Opin. Pulm. Med..

[4] Stone J., Hangge P., Albadawi H., Wallace A., Shamoun F., Knuttien M.G., Naidu S., Oklu R. (2017). Deep vein thrombosis: Pathogenesis, diagnosis, and medical management. Cardiovasc. Diagn. Ther..

[5] Caprini J.A. (2010). Risk assessment as a guide for the prevention of the many faces of venous thromboembolism. Am. J. Surg..

[6] Anderson D.R., Morgano G.P., Bennett C., Dentali F., Francis C.W., Garcia D.A., Kahn S.R., Rahman M., Rajasekhar A., Rogers F.B. (2019). American Society of Hematology 2019 guidelines for management of venous thromboembolism: Prevention of venous thromboembolism in surgical hospitalized patients. Blood Adv..

[7] Khaldi A., Helo N., Schneck M.J., Origitano T.C. (2011). Venous thromboembolism: Deep venous thrombosis and pulmonary embolism in a neurosurgical population. J. Neurosurg..

[8] Henwood P.C., Kennedy T.M., Thomson L., Galanis T., Tzanis G.L., Merli G.J., Kraft W.K. (2011). The incidence of deep vein thrombosis detected by routine surveillance ultrasound in neurosurgery patients receiving dual modality prophylaxis. J. Thromb. Thrombolysis.

[9] Rolston J.D., Han S.J., Bloch O., Parsa A.T. (2014). What clinical factors predict the incidence of deep venous thrombosis and pulmonary embolism in neurosurgical patients?. J. Neurosurg..

[10] Piper K., Algattas H., De Andrea-Lazarus I.A., Kimmell K.T., Li Y.M., Walter K.A., Silberstein H.J., Vates G.E. (2017). Risk factors associated with venous thromboembolism in patients undergoing spine surgery. J. Neurosurg. Spine.

[11] Chan A.T., Atiemo A., Diran L.K., Licholai G.P., McLaren Black P., Creager M.A., Goldhaber S.Z. (1999). Venous thromboembolism occurs frequently in patients undergoing brain tumor surgery despite prophylaxis. J. Thromb. Thrombolysis.

[12] Cote D.J., Dubois H.M., Karhade A.V., Smith T.R. (2016). Venous Thromboembolism in Patients Undergoing Craniotomy for Brain Tumors: A U.S. Nationwide Analysis. Semin. Thromb. Hemost..

[13] Dubinski D., Won S.Y., Voss M., Keil F., Miesbach W., Behmanesh B., Dosch M., Baumgarten P., Bernstock J.D., Seifert V. (2022). Direct oral anticoagulants vs. low-molecular-weight heparin for pulmonary embolism in patients with glioblastoma. Neurosurg. Rev..

[14] Ray W.Z., Strom R.G., Blackburn S.L., Ashley W.W., Sicard G.A., Rich K.M. (2009). Incidence of deep venous thrombosis after subarachnoid hemorrhage. J. Neurosurg..

[15] Serrone J.C., Wash E.M., Hartings J.A., Andaluz N., Zuccarello M. (2013). Venous thromboembolism in subarachnoid hemorrhage. World Neurosurg..

[16] Reiff D.A., Haricharan R.N., Bullington N.M., Griffin R.L., McGwin G., Rue L.W. (2009). Traumatic brain injury is associated with the development of deep vein thrombosis independent of pharmacological prophylaxis. J. Trauma.

[17] Schulman S., Kearon C. (2005). Definition of major bleeding in clinical investigations of antihemostatic medicinal products in non-surgical patients. J. Thromb. Haemost..

[18] Rethinasamy R., Alias A., Kandasamy R., Raffiq A., Looi M.C., Hillda T. (2019). Deep Vein Thrombosis and the Neurosurgical Patient. Malays. J. Med. Sci..

[19] Parmontree P., Ketprathum P., Ladnok T., Meeaium S., Thanaratsiriworakul T., Sonhorm U. (2022). Predictive risk factors for venous thromboembolism in neurosurgical patients: A retrospective analysis single center cohort study. Ann. Med. Surg..

[20] Liu X., Zhang X., Ma T., Li M., Zhang L., Li S., Zeng M., Kass I.S., Peng Y. (2023). Risk factors for postoperative thrombosis-related complications in patients undergoing malignant brain tumor resection: A retrospective cohort study. Front. Neurol..

[21] Kimmell K.T., Jahromi B.S. (2015). Clinical factors associated with venous thromboembolism risk in patients undergoing craniotomy. J. Neurosurg..

[22] Aishima K., Yoshimoto Y. (2013). Screening strategy using sequential serum D-dimer assay for the detection and prevention of venous thromboembolism after elective brain tumor surgery. Br. J. Neurosurg..

[23] Smith T.R., Nanney A.D., Lall R.R., Graham R.B., McClendon J., Lall R.R., Adel J.G., Zakarija A., Cote D.J., Chandler J.P. (2015). Development of venous thromboembolism (VTE) in patients undergoing surgery for brain tumors: Results from a single center over a 10 year period. J. Clin. Neurosci..

[24] Kaewborisutsakul A., Tunthanathip T., Yuwakosol P., Inkate S., Pattharachayakul S. (2020). Incidence and Risk Factors for Venous Thromboembolism Following Craniotomy for Intracranial Tumors: A Cohort Study. Asian J. Neurosurg..

[25] Chow J.H., Chancer Z., Mazzeffi M.A., McNeil J.S., Sokolow M.J., Gaines T.M., Reif M.M., Trinh A.T., Wellington I.J., Camacho J.E. (2021). Impact of Preoperative Platelet Count on Bleeding Risk and Allogeneic Transfusion in Multilevel Spine Surgery. Spine.

[26] Pang C.H., Lee S.E., Kim C.H., Kim J.E., Kang H.S., Park C.K., Paek S.H., Kim C.H., Jahng T.A., Kim J.W. (2015). Acute intracranial bleeding and recurrence after bur hole craniostomy for chronic subdural hematoma. J. Neurosurg..

[27] Liu D., Song D., Ning W., Zhang X., Chen S., Zhang H. (2023). Efficacy and safety of prophylaxis for venous thromboembolism in brain neoplasm patients undergoing neurosurgery: A systematic review and Bayesian network meta-analysis. J. Thromb. Thrombolysis.

[28] Tomlinson S.B., Piper K., Kimmell K.T., Vates G.E. (2017). Preoperative Frailty Score for 30-Day Morbidity and Mortality After Cranial Neurosurgery. World Neurosurg..

[29] Forgerini M., Varallo F.R., Oliveira A.R.A., Nadai T.R., Mastroianni P.C. (2019). Assessment of the adherence to and costs of the prophylaxis protocol for venous thromboembolism. Clinics.

[30] Lara-Reyna J., Alali L., Wedderburn R., Margetis K. (2022). Compliance with venous thromboembolism chemoprophylaxis guidelines in non-operative traumatic brain injury. Clin. Neurol. Neurosurg..

[31] Choucha A., Troude L., Morin L., Fernandes S., Baucher G., De Simone M., Lihi A., Mazen K., Alseirihi M., Passeri T. (2024). Management of large Trigeminal Schwannoma: Long-term oncologic and functional outcome from a multicentric retrospective cohort. Acta Neurochir..

